# Achieving Real-Time Prediction of Paroxysmal Atrial Fibrillation Onset by Convolutional Neural Network and Sliding Window on R-R Interval Sequences

**DOI:** 10.3390/bioengineering11090903

**Published:** 2024-09-10

**Authors:** Wenjing Chen, Peirong Zheng, Yuxiang Bu, Yuanning Xu, Dakun Lai

**Affiliations:** 1 West China Hospital, Sichuan University, Chengdu 610041, China; chenwenjing0626@scu.edu.cn; 2 School of Electronic Science and Engineering, University of Electronic Science and Technology of China, Chengdu 611731, China; peirong.zheng@connect.polyu.hk (P.Z.); 18827668487@163.com (Y.B.); 3 Department of Computing, The Hong Kong Polytechnic University, Hong Kong SAR, China

**Keywords:** arrhythmias, paroxysmal atrial fibrillation, electrocardiogram, heart rate variability, deep learning, onset prediction, real time, monitoring, algorithm

## Abstract

Early diagnosis of paroxysmal atrial fibrillation (PAF) could prompt patients to receive timely interventions in clinical practice. Various PAF onset prediction algorithms might benefit from accurate heart rate variability (HRV) analysis and machine learning classification but are challenged by real-time monitoring scenarios. The aim of this study is to present an end-to-end deep learning-based PAFNet model that integrates a sliding window technique on raw R-R intervals of electrocardiogram (ECG) segments to achieve a real-time prediction of PAF onset. This integration enables the deep convolutional neural network (CNN) to be customized as a light-weight architecture that accommodates the size of sliding windows simply by altering the input layer, and specifically its effectiveness in making a new prediction with each new heartbeat. Catering to the potential influence of input sizes, three CNN models were trained using 50, 100, and 200 R-R intervals, respectively. For each model, the performance of the automated algorithms was evaluated for PAF prediction using a ten-fold cross-validation. As a results, a total of 56,381 PAFN-type and 56,900 N-type R-R interval segments were collected from publicly accessible ECG databases, and a promising prediction performance of the automated algorithm with 100 R-R intervals was achieved, with a sensitivity of 97.12%, a specificity of 97.77%, and an accuracy of 97.45%, respectively. Importantly, the automated algorithm with a sliding window step of 1 could process one sample in only 23.1 milliseconds and identify the onset of PAF at least 45 min in advance. The present results suggest that the sliding window technique on raw R-R interval sequences, along with deep learning-based algorithms, may offer the possibility of providing an accurate, real-time, end-to-end clinical tool for mass monitoring of PAF.

## 1. Introduction

Atrial fibrillation (AF) is a commonly sustained arrhythmia in clinical practice which can significantly impair the quality of life and increase the risk of serious medical conditions, including stroke and heart attack [1]. The prevalence of AF increases with age [2]; therefore, as the aging population problem becomes increasingly prominent, the threat of atrial fibrillation to human health becomes increasingly severe. When AF occurs, the disorganized fibrillation of the atrium will reduce the cardiac output and accelerate the formation of a thrombus, which may cause blood vessel block and further lead to life-threatening diseases such as ischemic stroke [3] and myocardial infarction [4].

To evaluate the risk of AF during different phases and undertake intervention and treatment in time, AF is commonly divided into different types. Currently, AF classification according to the presentation, duration, and spontaneity of AF episodes has become a consensus in authoritative guidelines [5]. The corresponding five AF types are as follows: first-diagnosed AF, paroxysmal AF (PAF), persistent AF, long-standing persistent AF, and permanent AF. At the beginning, AF usually manifests as PAF, which is defined as AF that terminates spontaneously or with intervention within 7 days of onset. There are many ways to manage and treat AF, including drug therapy, implanted medical instruments, and radio frequency ablation, but all these methods carry potential risks. For example, drug therapy has been shown to be effective in patients with newly diagnosed AF with a treatment success rate of about 50% [6,7]. But in the case of patients with persistent AF, it may not only be less effective but also cause other arrhythmias and even fatal complications. So, to prevent irreversible atrial lesions and prevent the further deterioration of AF, early diagnosis of AF has become particularly important.

Electrocardiogram (ECG) is a commonly used tool that could assist cardiologists in diagnosing AF in clinical practice. The development of accurate predictors based on ECG is important for designing high-performance models [5]. There are three types of ECG episodes seen in both normal and PAF subjects, and they can be divided as follows: (A) ECG episode of a normal subject in resting state, (B) ECG episode of a PAF subject when AF does not occur, and (C) ECG episode of a PAF subject when AF occurs. Compared with an ECG episode of a normal subject, ECG episode (B), recorded in PAF subjects when AF does not occur, occasionally has premature beats and subtle changes in R-R interval, which could be used as predictors of the onset of PAF. As such, developing a PAF onset prediction model would be significant for several reasons. First, when AF does not occur, it is difficult to distinguish the ECG of PAF subjects from that of normal subjects. An automatic PAF onset prediction model could assist clinicians in the risk assessment of patients with PAF. Second, a positive prediction result could help patients to receive timely interventions, like drug therapy, which could effectively prevent the deterioration of AF in PAF subjects. Third, during postoperative follow-up of radio frequency ablation surgery, such a model would also be helpful in assessing the surgical effect [7].

In recent decades, many PAF onset prediction algorithms based on machine learning methods have been proposed, with most of these being based on the extracted heart rate variability (HRV) feature, including time domain, frequency, and non-linear and time-frequency domain features. In March 2001, the PhysioNet Computing in Cardiology Challenge 2001 was held [8], during which researchers proposed various methods to predict the onset of PAF, such as methods based on HRV features [9,10,11,12], atrial premature contraction numbers [13,14], rhythm-based heartbeat duration normalization [15], and P-wave morphology [16]. The publicly accessible PAF prediction challenge database (AFPDB), which could be used to train and test the classification model, was also provided in this competition. Recently, Mohebbi [17] extracted spectrum, bispectrum, and non-linear features from the 30 min HRV signal and used a support vector machine (SVM)-based classifier to predict the onset of PAF, achieving a sensitivity of 96.3%. Boon [18] used a genetic algorithm to optimize features extracted from a 15 min HRV signal and also used an SVM classifier to predict the onset of PAF, achieving an accuracy of 79.3%. In another study, they used a shorter 5 min HRV signal and achieved an accuracy of 87.7% [19]. Narin [20] also used a 5 min HRV signal for linear and non-linear feature extraction; they used a k-nearest neighbors (KNN) classifier and further discussed the performance of the model for data segments in different time windows. Wang [21] improved the speed of the SVM algorithm and achieved an accuracy of 92.5% for the test set of different databases, but the required length of the signal was 5 min long, and the generalization ability (87.0% accuracy) in clinical tests was unsatisfying. Sutton [22] proposed the PhysOnline, an open-source streaming physiological signal analysis platform, and demonstrated the effective online prediction of PAF. Although HRV analysis, commonly used in such studies on the topic of PAF prediction, could be compatible with feature selection methods and machine learning classifiers, the extraction and selection of hand-crafted features would be an inevitably subjective, time-consuming, and labor-intensive process. Most recently, some studies have reported on developing AF detection algorithms using deep learning methods [23,24,25,26,27], which show better performance compared with feature extraction [28,29,30] and machine learning methods [31,32,33]. However, few studies on the topic of PAF prediction based on deep learning methods have been presented so far, and a prominent limitation of these machine learning-based methods is their poor real-time performance, since the time duration of the ECG signal used for HRV analysis is commonly at least a couple of minutes, which does not meet the requirements of real-time monitoring scenarios [34].

To address these challenges, this paper proposes a novel deep learning-based method for real-time PAF onset prediction, named the PAFNet model. This automated algorithm integrates a sliding window technique on raw R-R intervals of ECG segments with an end-to-end convolutional neural network (CNN). This integration enables the CNN model to accommodate the size of the sliding windows by only altering the input layer, and specifically its effectiveness in making a new prediction with each new heartbeat. This algorithm aims to mitigate the limitations of traditional PAF prediction methods: vulnerability to subjectivity, poor real-time performance, and limited contextual understanding. Our experiments on a variety of publicly accessible ECG databases show that our algorithm improves the accuracy and real-time performance of PAF prediction. The contributions of this paper are as follows:

(1) We propose a novel automated algorithm for real-time PAF onset prediction which uses a sliding window on raw R-R intervals of ECG segments. This mechanism allows the model to easily adjust the sliding step to meet different application scenarios. We set the sliding step to 1 in this study to meet real-time monitoring requirements.
(2) We also introduce a CNN model for end-to-end PAF prediction and classification with only raw R-R interval segments as input samples, which allows the whole system to automatically emphasize important information in the input data and avoid the inevitable subjectivity of using machine learning methods.
(3) By comparing the results produced with different input sizes of the model, we found that 100 R-R intervals resulted in an overall improvement in prediction performance, and 50 and 200 R-R intervals were relatively less efficient in terms of the testing time of each sample.
(4) We carried out comprehensive and comparative experiments using public datasets to validate the effectiveness of our model. The results demonstrate that our approach performs exceptionally well in PAF prediction tasks and holds promise for real-time applications.

The rest of the article is structured as follows. In Section 2, the databases and detailed methods are both presented. The methods are evaluated in Section 3. Section 4 discusses the analysis and results. Finally, Section 5 concludes this article by summarizing the achievements and stating possible future applications.

## 2. Materials and Methods

### 2.1. Databases

Table 1 shows that we used AFPDB for training and validating the PAFNet, while the MIT-BIH Atrial Fibrillation Database (AFDB) and the MIT-BIH Normal Sinus Rhythm Database (NSRDB) were used to test the model’s performance and generalization ability. These publicly accessible databases are available from PhysioNet [34] and contain two ECG channels. As all channels were collected simultaneously and possess the same R-R interval information, we used only single-lead ECG to derive the R-R interval sequence.

**Figure 1:**
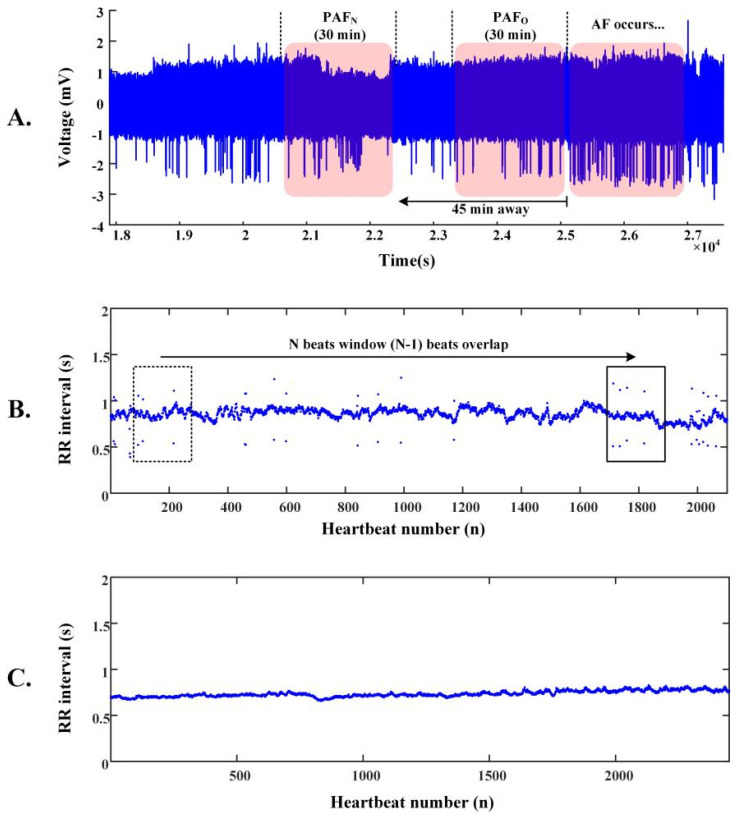
Demonstration of the data segmentation used for training PAFNet. (**A**) Two types of 30 min ECG records before the onset of AF are shown: PAF normal (PAFN) type, which is at least 45 min away from any AF episodes, and PAF onset (PAFO) type, which is just before the onset of AF. (**B**) An example of an R-R interval sequence derived from a PAFN record and the corresponding data segmentation based on the sliding window technique. (**C**) An example of an R-R interval sequence derived from a normal sinus record.

Figure 1A shows the learning set of AFPDB, which contains three types of labeled ECG records: PAF normal (PAFN) type, which is at least 45 min away from any AF episodes; PAF onset (PAFO) type, which is just near the onset of AF; and normal (N) type, with each record lasting 30 min. To predict the onset of PAF at least 45 min in advance, we used 25 PAFN-type ECG records and 25 N-type ECG records.

For the test databases, the AFDB includes 25 long-term ECG records from subjects with AF (mostly PAF), and the NSRDB includes 18 long-term ECG records from subjects with no significant arrhythmia. We extracted PAFN-type records from the AFDB using the same protocol as the AFPDB, excluding AF segments less than 5 min, atrial flutter segments, and atrial ventricular junction rhythm segments. As a result, we extracted 12 PAFN-type ECG records and 18 N-type ECG records from these two databases, with each record lasting 30 min.

### 2.2. R-R Intervals of ECG Segments

Pre-processing of ECG records through filtering is crucial for improving signal quality and R-wave location accuracy. To reduce different types of noise interferences, we adopted a series of digital filters. First, we used a band-pass filter with a cutoff frequency of 0.1 Hz to 100 Hz to filter out noise beyond the useful frequency range. Next, we removed the baseline drift using a median filter with a window size set to 0.85 of the sampling frequencies. Finally, we used a fourth-order low-pass filter to further eliminate high-frequency noise.

Figure 1B,C demonstrate the data segmentation procedure. The R-R interval sequence of PAFN subjects is more fluctuant than that of normal subjects. After pre-processing, we accurately located the R-waves of each ECG record using the threshold difference algorithm, and derived the R-R interval sequence using this equation:

(1) $$RR_{i}=R_{i+1}-R_{i}$$

where *RR_i_* represents the value of the i-th R-R interval, *R_i_* represents the time index of the i-th R-wave, and the index *i* ranges from 1 to M when the ECG record contains (M + 1) R-waves.

We then adopted a sliding window with a size of N on each R-R interval sequence. This window continuously moved from one side to another and derived a segment containing N R-R intervals during each move. The sliding step could be adjusted according to different application scenarios. In this study, we set the sliding step to 1 to meet the real-time processing requirements and the massive amount of data required for training deep learning models. With a sliding step of 1, we derived (M − N + 1) R-R interval segments from the whole R-R interval sequence.

### 2.3. Architecture of the PAFNet Model

In this study, we explored a real-time and accurate method for predicting the onset of PAF at least 45 min in advance by developing a 1D CNN model. Unlike methods that rely on manually extracted HRV features and traditional machine learning classifiers, end-to-end deep learning techniques avoid the need for hand-crafted feature extraction, thus reducing the loss of ECG information and the limitations of prior knowledge. Among these techniques, CNN is well-suited for image processing and automatic feature extraction, making it ideal for image classification and identification [35]. Similarly, the ECG signal and R-R interval sequence contain abundant overall and partial information that can be automatically extracted using CNN to identify specific diseases. The CNN model can extract high-level feature maps from 1D signal, enabling accurate identification of specific patterns related to the onset of PAF.

**Figure 2:**
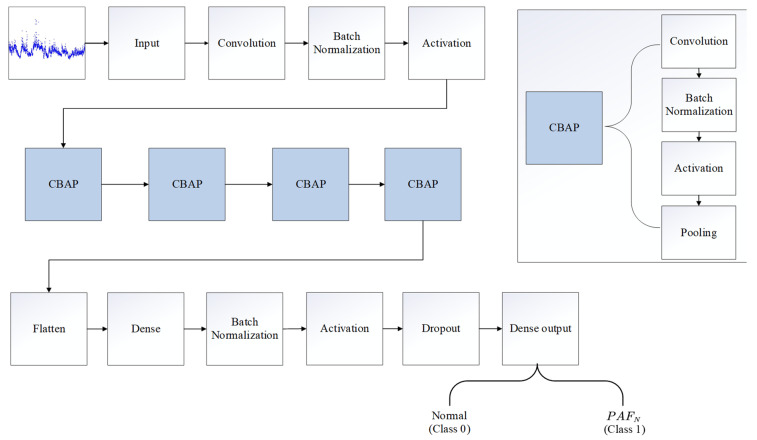
Illustration of the model structure of the proposed CNN model, named as PAFNet in this study. The 1D convolutional layer, batch normalization layer, activation layer, and 1D max pooling layer are abstracted as a CBAP layer.

As shown in Figure 2, PAFNet consisted of 26 layers, including 5 convolutional layers. The input size is the same as the sliding window, with each sample represented by a matrix of one row and N columns, where the column number is the index of the R-R interval, and the value is the corresponding R-R interval duration in seconds. The 1D convolutional layer, batch normalization layer, activation layer, and 1D maximum pooling layer were abstracted as a block CBAP layer. The convolutional layer automatically extracted feature maps using kernel techniques, while the batch normalization layer accelerated training and improved accuracy. The activation layer increased the non-linearity of the model, and the pooling layer reduced the scale of the feature map. The flatten layer converted all feature maps into one row as input to the dense layer for the final prediction. The output represents the binary prediction result of PAFNet.

Table 2 displays the details of the PAFNet’s architecture, including hyperparameters and activation functions used. The size of the input layer depends on the size of the sliding window, and the size of the output layer is set to 1, which represents the probability that the corresponding sample is PAFN. For the CBAP layer, a convolutional kernel size of 100 and a stride of 16 were selected, and the padding method was set to ‘valid’. The ReLU function was used as the activation function. A pooling kernel size of 2 and a stride of 2 were selected to halve the scale of each feature map. The number of output feature maps was set to 16, 32, 64, and 128 for the four CBAP layers, respectively. The size of the flatten layer also depends on the size of the sliding window, and the node number of the first dense layer is set to 2,048. A dropout ratio of 0.5 was used to randomly deactivate half of the nodes during each iteration. The training epoch was set to 9, and the batch size was set to 512.

### 2.4. Training and Optimization of the PAFNet Model

After determining the model’s structure, the next step was to optimize the size of the sliding window. Three types of evaluation metrics were used to assess the model’s performance with different input sizes, including testing time per batch. A small input size may result in decreased performance due to insufficient ECG information captured by the sliding window, while a larger input size includes more details but may require more testing time per batch.

The limited total sample number of the databases necessitated the use of a stratified ten-fold cross-validation strategy to optimize and evaluate the performance of the PAFNet model during the training and testing procedures. The training dataset, with a sliding window size of 100, yielded 113,281 R-R interval segments, consisting of 56,381 PAFN type and 56,900 N type R-R interval segments. To train PAFNet, all segments were randomly divided into ten parts, with nine parts used for training and one part used for validation. This resulted in ten CNN models being trained and saved, with the prediction result of PAFNet during the testing procedure obtained by averaging the prediction result of these models. The model’s performance was evaluated using the receiver operator characteristic (ROC) curve, which compared the prediction results obtained when using samples of different spans before the onset of PAF as input.

### 2.5. Evaluation Protocols

The ability of the PAFNet to predict the onset of PAF was evaluated quantitatively using sensitivity (*Sen*), specificity (*Spe*), and accuracy (*Acc*). The total number of true positives (*TPs*), false negatives (*FNs*), true negatives (*TNs*), and false positives (*FPs*) was counted for PAFN type as positive and N type as negative, and *Sen*, *Spe*, and *Acc* were calculated based on these statistical parameters.

Finally, based on these statistical parameters, *Sen*, *Spe*, and *Acc* were calculated as follows:

(2) $$Sen=\frac{TP}{TP+FN}$$

(3) $$Spe=\frac{TN}{TN+FP}$$

(4) $$Acc=\frac{TP+TN}{TP+FN+TN+FP}$$

## 3. Results

In this study, the training and testing of the PAFNet model were conducted using the TensorFlow 2.3.0 deep learning framework on a desktop computer equipped with an Intel(R)Core(TM)i9-10900KF CPU@3.70 GHz and 64 GB memory. To accelerate processing and reduce training and testing time, an NVIDIA GeForce RTX 3080 GPU with 10 GB memory was also utilized.

Table 3 summarizes the results of the model input size optimization. Three models were trained using input sizes of 50, 100, and 200 R-R intervals, denoted as M1, M2, and M3, respectively. The total parameter count is in the range of millions. The testing results of these models show that M2 achieved the highest Sen, Spe, and Acc, with values of 89.92%, 93.24%, and 91.96%, respectively. Notably, M2 exhibited a 4% increase in Sen and nearly a 1% increase in Spe compared to M1 and M3. Accordingly, the Acc of M2 increased by nearly 2%, indicating an overall improvement in the model’s performance. In terms of testing time, M2 was the most efficient, taking only 9.3 milliseconds to process one input sample, whereas M1 and M3 took 13.8 milliseconds and nearly 30 milliseconds, respectively, to process a batch of data (i.e., 512 samples). Based on these results, an input size of 100 was selected, and M2 was identified as the optimized model.

Table 4 presents the results of ten-fold cross-validation of the PAFNet model using 100 R-R intervals as input (M2 in Table 3). The sliding window size is set to 100, with a sliding step of 1 as mentioned in Section 2. During the ten-fold cross-validation, the 113,281 training and validation samples are randomly shuffled and divided into 10 parts, with each part used once for validation and nine times for training. The fifth and seventh folds show the highest accuracy of 100.00%, while the first fold shows the lowest accuracy of 87.16%, indicating significant variability. Notably, the average validation results are substantially higher than the testing results of *Sen*, *Spe*, and *Acc* for M2 in Table 3, at 97.12%, 97.77%, and 97.45%, respectively.

**Figure 3:**
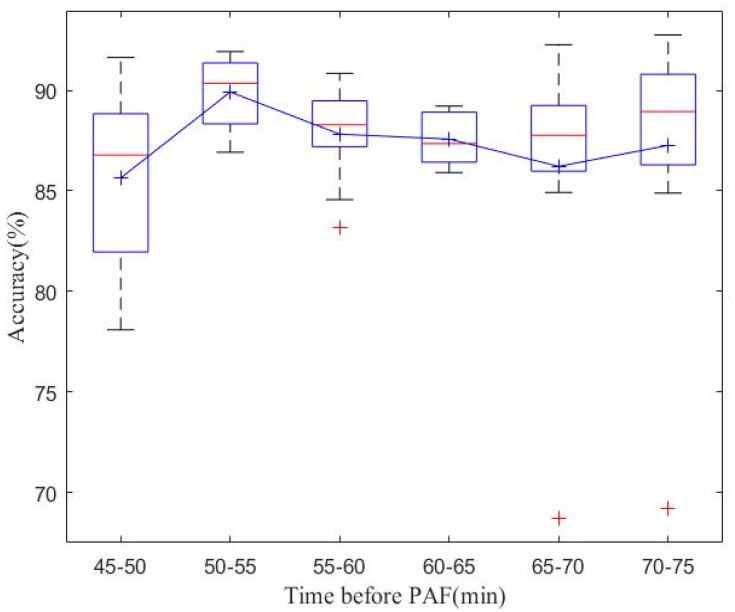
Comparison of model classification accuracy using ECG signals in different time periods before onset of AF as input data of PAFNet. Explanation for the notations: (a) Red line: the median value for each time interval; (b) Red +: outliers; (c) Blue square: the bottom and top edges represent the first and third quartiles; (d) Black dash lines: to show the rest of the distribution, excluding outliers. (e) Blue +: the mean value.

**Figure 4:**
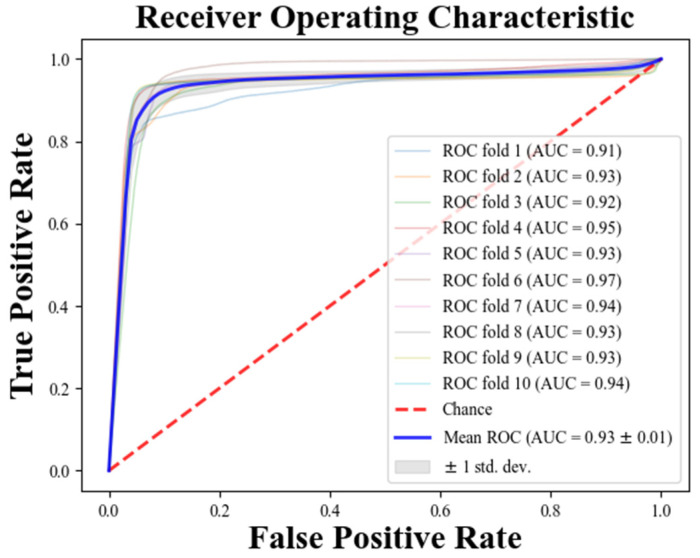
Receiver operating characteristic (ROC) curves of the ten folds during the model test.

Figure 3 and Figure 4 present the results of database-level testing to evaluate the generalization ability of the PAFNet model. Figure 3 shows the prediction accuracy of the trained PAFNet using AFPDB, tested using the databases AFDB and NSRDB. Additionally, the evaluation includes input samples of different spans before the onset of PAF. The horizontal axis represents the different spans, which started 75 min before the PAF onset, while the vertical axis represents the prediction accuracy. The resultant curve indicates that the accuracy fluctuates around 85% and does not significantly change when the span of the sample varies. Figure 4 depicts the receiver operator characteristic (ROC) curves of the ten models during the ten folds of the stratified ten-fold cross-validation. The bold blue curve denotes the ROC curve of the average prediction results. The proposed PAFNet achieved high performance for both positive and negative samples, and the mean area under the curve (AUC) is about 0.93, with AUC values ranging from 0.91 to 0.97 in each fold.

## 4. Discussions

### 4.1. Real-Time PAF Onset Prediction

HRV analysis is commonly used in studies on the topic of PAF prediction. Extracted HRV features, including time domain, frequency, non-linear, and time-frequency domain features, could reflect the variability of R-R intervals and indirectly reflect the influence of premature beats and other heart rhythms. This kind of method has a relatively mature theoretical system and implementation and could be compatible with feature selection methods and machine learning classifiers, but the time duration of the ECG signal used for HRV analysis is at least 5 min, which does not meet the requirements of real-time monitoring scenarios [36].

Unlike HRV analysis, we proposed a real-time PAF onset prediction method based on raw R-R interval sequences and the sliding window technique, which effectively solves the problem of poor real-time performance in traditional PAF prediction methods. Compared with the commonly used HRV analysis, which requires at least a 5 min ECG signal, our method allows for real-time monitoring scenarios such as ICU PAF monitoring. The sliding window technique enables the capture of R-R intervals at a fixed number as the window moves on the R-R interval sequence, updating with each new R wave detected by the R wave location algorithm. The highly effective PAF prediction algorithm, which processes one sample in only 23.1 milliseconds, allows the whole system to make a new prediction with each new heartbeat, meeting the requirements of real-time PAF onset prediction scenarios. This method offers a promising approach for real-time PAF prediction, with potential for further development and application in clinical settings. 

### 4.2. Performance Compared with Other Methods

Our proposed method for predicting PAF offers several advantages, including the use of deep learning to improve prediction accuracy. We used the CNN technique to achieve end-to-end prediction and classification, with raw R-R interval segments as input samples to preserve ECG information. PAF prediction requires longer ECG signals than AF detection or classification, as shown in Table 3. When the input size was set to 50 (approximately 30–50 s ECG signal), the prediction accuracy was 89.74%. Increasing the input size to 100 did not significantly improve accuracy, which was 91.42%, approximately equal to the accuracy achieved with an input size of 200. However, increasing the input size results in a smaller total number of samples, which is why deep learning is rarely used in related studies. To address this issue, we adopted a sliding window with a step of one R-R interval, generating a sufficient training database. Using this methodology, we achieved high accuracy in predicting PAF and demonstrated the effectiveness of our approach.

Table 4 shows the benchmarking results of our proposed method against other previous studies. Our PAFNet performed better than most studies using machine learning methods. These studies used input sizes varying from 5 to 30 min to extract different HRV features and employed classifiers such as SVM and KNN to predict PAF. Compared to these studies, our proposed PAFNet achieved higher *Sen*, *Spe*, and *Acc* than studies using input sizes of 5 and 15 min [3,5,6]. However, the study by Mohebbi [17] showed better performance than ours, likely due to their use of the whole 30 min ECG from each record, which sacrificed real-time performance for improved prediction accuracy. Additionally, their studies did not conduct database-level tests. Wang [8] developed models with competitive performance on testing, but their model requires a 5 min input and a hand-crafted P wave, which is significantly longer than the input length required for our proposed method. Overall, our proposed PAFNet demonstrates the effectiveness of a deep learning approach, outperforming most previous studies using machine learning methods.

### 4.3. Study Limitations and Future Works

Although this study proposed a real-time PAF prediction model that outperformed many previous studies, there are still some limitations. Firstly, while the total number of R-R interval samples is enough for training the PAFNet model and the demonstrated competitive performance outperforms most previous studies validated in the same publicly accessible database of AFPDB, the number of ECG records of patients is limited, resulting in insufficient variation in the ECG signals and a fluctuation in the performance in the inter-database testing. It indicates that the generalization ability of the PAFNet could be further improved with a larger number of patients in the future. Secondly, all the programs were deployed on a desktop computer with an efficient GPU in this study. Future studies could focus on hardware implementation to test the real-time performance of the model on embedded systems such as smart watches or other mobile devices with limited resources and processing performance. Making the PAFNet lighter could be achieved by reducing the number of blocks and the size of the sliding window, as well as by increasing the step length. Thirdly, although this retrospective study presented an engineering investigation of the feasibility and accuracy of the proposed PAFNet model in PAF prediction with offline public ECG databases and clearly outperformed other similar studies in terms of both real-time processing speed and prediction performance, it was not a prospective, multicentered, and large-scale cohort study in a clinical setting. It would be interesting for future works to explore the potential use of this method in the aging population or in patients with other cardiovascular diseases, such as hypertension, or in specific clinical practices, such as predictions of AF relapse after first-time catheter ablation [37] or PAF after acute ischemic stroke [38], where patients are often underdiagnosed due to the challenging and resource-intensive diagnosis.

## 5. Conclusions

The capacity to predict PAF at least 45 min in advance can help clinicians assess the risk of PAF events and provide timely intervention for patients. However, previous PAF prediction studies based on HRV analysis and traditional machine learning methods have had limitations, such as unsatisfactory real-time processing speed and prediction performance. This study proposes a novel deep learning-based PAFNet model that incorporates the sliding window to achieve real-time prediction of PAF onset. This integration enables the CNN model to adapt to the size of the sliding windows simply by altering the input layer, specifically its effectiveness in making a new prediction with each new heartbeat. The model only requires a single-lead ECG signal as input to extract the R-R interval sequence, making it suitable for clinical application even in scenarios with limited medical resources and conditions. Our results suggest that the customized lightweight 26-layer CNN, with 5 weighted layers paired with a sliding window of a length of 100 R-R intervals, can achieve relatively high performance and may offer a real-time, accurate, and inexpensive clinical tool for predicting PAF events. The restricted nature of medical resources, such as 24 h bedside monitors in the intensive care unit or 24 h or long-term ECG Holter monitoring, requires our further research to be compatible with clinical practice by using a simpler ECG record, highlighting the opportunity to further to revalidate the performance and applicability of the PAF prediction model. To sum up, the PAFNet offers a promising way to enhance PAF prediction performance and has the potential to be a valuable tool for clinicians in identifying and treating PAF events.

## Figures and Tables

**Table 1:** ECG datasets.

Database		Number of Records (*n*)	Number of R-R Intervals (*n*)
Training and validation	AFPDB (PAFN)	25	56,381
	AFPDB (N)	25	56,900
Testing	AFDB (PAFN)	12	27,836
	NSRDB (N)	18	44,087

**Table 2:** The architecture of the proposed PAFNet.

Number	Layer Type	Number of Feature Maps or Nodes	Parameters	Number	Layer Type	Number of Feature Maps or Nodes	Parameters
1	input	changing with the size of the sliding window	N	14	BN *	-	-
2	convolutional	16	size: N, kernel: 8, padding = “same”	15	activation	-	ReLU
3	BN	-	-	16	pooling	-	size: 2
4	activation	-	ReLU	17	convolutional	256	size: N/16, kernel: 8, padding = “same”
5	convolutional	32	size: N/2, kernel: 8, padding = “same”	18	BN	-	-
6	BN	-	-	19	activation	-	ReLU
7	activation	-	ReLU	20	pooling	-	size: 2
8	pooling	-	size: 2	21	Flatten	-	-
9	convolutional	64	size: N/4, kernel: 8, padding = “same”	22	Dense	512	-
10	BN	-	-	23	BN	-	-
11	activation	-	ReLU	24	activation	-	ReLU
12	pooling	-	size: 2	25	dropout	-	0.25
13	convolutional	128	size: N/8, kernel: 8, padding = “same”	26	Dense output	1	activation function: Sigmoid

* BN = batch normalization.

**Table 3:** Results of three models with different input sizes of R-R intervals.

Model	Input Size (*n*)	*Sen* (%)	*Spe* (%)	*Acc* (%)	Testing Time (ms/batch)	Total Params
M1	50	85.44	92.45	89.74	13.8	878,017
M2	100	89.92	93.24	91.96	23.1	1,271,233
M3	200	88.17	93.47	91.42	43.0	2,057,665

**Table 4:** Results of ten-fold cross-validation using an overlapped window of 100 R-R intervals as input.

Fold	Training Data (Rows)	Validation Data (Rows)	*Sen* (%)	*Spe* (%)	*Acc* (%)
1	11,329–113,281	1–11,328	82.11	92.09	87.16
2	1–11,328, 22,656–113,281	11,328–22,656	95.34	87.84	91.63
3	1–22,656, 33,984–113,281	22,656–33,984	98.74	98.86	98.80
4	1–33,984, 45,312–113,281	33,984–45,312	99.39	99.39	99.39
5	1–45,312, 56,640–113,281	45,312–56,640	100.00	100.00	100.00
6	1–56,640, 67,968–113,281	56,640–67,968	98.76	99.95	99.35
7	1–67,968, 79,296–113,281	67,968–79,296	100.00	100.00	100.00
8	1–79,296, 90,624–113,281	79,296–90,624	98.47	100.00	99.21
9	1–90,624, 101,952–113,281	90,624–101,952	98.43	99.54	98.98
10	1–101,952	101,952–113,281	100.00	100.00	100.00
Mean	-	-	97.12	97.77	97.45
Var *			0.0030	0.0018	0.0019

* Var = variance.

## Data Availability

The MIT-BIH Atrial Fibrillation Database (AFDB) and the MIT-BIH Normal Sinus Rhythm Database (NSRDB) are publicly accessible from PhysioNet https://www.physionet.org/about/database/ (accessed on 20 March 2021).
